# A Proposed Scale to Assess Magnesium Status Using Serum Calcium and Magnesium Ratios

**DOI:** 10.3390/nu17233671

**Published:** 2025-11-24

**Authors:** Andrea Rosanoff, Bodo von Ehrlich, Deanna Nelson

**Affiliations:** 1 CMER Center for Magnesium Education & Research, Pahoa, HI 96778, USA; 2 Internal Medicine Private Practice, 87435 Kempten, Germany; aioloskalo@t-online.de; 3 BioLink Life Sciences, Inc., Cary, NC 27513, USA; dnelson@phosveda.com

**Keywords:** magnesium assessment scale, serum Mg/Ca, serum Ca/Mg, serum magnesium–calcium ratios, serum calcium-magnesium ratios, magnesium, calcium, nutrient, physiological magnesium deficit, magnesium assessment

## Abstract

**Background/Objectives:** Reliable markers of human magnesium (Mg) status are needed. **Methods:** Current Mg studies report ratios between serum Mg and calcium (Ca) using four interchangeable expressions (i.e., molar or weight calculations of Mg/Ca or Ca/Mg). We propose a scale using ratios of serum Mg and Ca to assess Mg status, unified for all four expressions. We explore its application for case studies and published research. **Results:** *Case Studies (4)*—the proposed serum Mg/Ca scale showed better Mg diagnostic value than serum Mg alone. *Published Studies*—A. The proposed Mg/Ca scale’s “depleted Mg” status predicted mortality among hospitalized COVID-19 patients in ROC/AUC analyses. B. The serum Ca/Mg proposed scale, when applied to “healthy” adults with “normal” serum Mg, exposed a “seriously depleted” to “adequate” range of Mg status. C. In a study of periodontal disease, patients designated “adequate” or “mild” Mg depletion by the proposed scale showed greater 5-year improvement than those with scale’s “moderate to serious” Mg depletion status. D. Finally, the proposed scale demonstrated appropriate diagnostic value of serum Ca/Mg in acute coronary syndrome patients only when “corrected Ca” was NOT used in ratio calculation. **Conclusions:** The proposed scale needs both total serum Ca and Mg measures in identical units, i.e., mg/dL or mg% (for weight ratios); mmol/L or mEq/L (for molar ratios). Authors/reviewers/editors need to take care when reporting units/methodology of serum Mg and Ca ratios for clear reporting as to weight or molar ratio and use of total or corrected values. Future trials and statistical testing are needed to determine whether ratios between serum Mg and Ca yield more precise measures of physiological Mg status than serum Mg alone. Our findings indicate the proposed scale is worthy of further study as a marker of Mg deficit.

## 1. Introduction

The global magnesium (Mg) research community has long sought a reliable, reproducible, and simple measure of Mg status in humans for both research and clinical practice settings [1,2,3,4,5]. Although Mg status (both dietary and blood levels) is consistently associated with several chronic disease states in humans [6,7,8,9,10,11,12], a reliable, precise, convenient marker of individual human Mg status has remained elusive. This gap has meant that several individuals with an asymptomatic Mg deficit do not receive a diagnosis or Mg therapy, enhancing their risk of developing chronic diseases associated with suboptimal Mg status [4,6].

Consumption of the modern processed food diet, which is low in Mg [13], is increasing globally [14]. This modern processed food diet is high in refined grains, refined sugars, and refined oils, resulting in Mg losses of 80–100% in processed foods compared with their unrefined plant sources [15]. Higher levels of dietary Mg inadequacy have been observed in both national and global populations [16,17], with pregnant females, adolescents, and older adults having the highest risk of Mg deficiency [13].

Mg homeostasis is now known to include three physiological processes in the regulation of serum Mg: (1) the Mg absorption level in the gut; (2) the Mg reabsorption level in the kidneys; and (3) the transfer of Mg from body stores, with the most important tissues being bone and muscle [18]. Thus, when gastrointestinal absorption and kidney reabsorption of Mg are both at their physiologically adapted maximum, translocation of Mg body stores can come into play [19]. It is safe to assume that patients with suboptimal Mg status are in an active physiological process of body-store Mg depletion and thus in need of Mg therapy, as several disease states are associated with a low Mg status [13,20,21,22]. Therefore, it is incumbent on the medical community to reliably assess Mg status to enable proper nutritional, therapeutic, and preventive medicine by using the inexpensive, generally safe Mg therapies when warranted. The use of serum Mg alone or dietary Mg assessment both fall short for the diagnosis of Mg deficiency, and a reliable, inexpensive, widespread Mg assessment tool is needed.

Dietary Mg is not only time-consuming to assess, but it is also imprecise because it relies on food tables that represent overall means of mineral concentrations from foods that vary widely, given the location, season, weather, soil conditions, and cultivars [13]. Mg load testing or assessment of urinary Mg concentration can be reliable [23] but cumbersome, with both requiring 24 h urine collection. Clinical tests for serum Mg concentrations are widely available and relatively inexpensive, but they are rarely used as a measure of Mg status because serum Mg alone can be unreliable. In addition, reference range cutoffs for serum Mg for defining hypomagnesemia vary widely [24]. Owing to these myriad serum Mg reference ranges, nonstandardized hypomagnesemia cutoff values are often found to miss patients who are, in fact, Mg-deficient [25,26,27] (see the Discussion in Section 5).

We suggest that the ratio between serum Mg and serum calcium (Ca) may be a more precise measure of Mg status than serum Mg alone. Measures of serum Mg and Ca are noninvasive, widely available, and inexpensive, and their ratio is easily calculated.

The purpose of this review is to introduce a proposed scale for Mg status using the ratio between serum Ca and serum Mg (hereinafter, proposed scale). This proposed scale was developed over the past two decades in a private medical practice (Appendix B). We first present four patient case studies using the proposed scale, and then we assess the proposed scale’s applicability to research by reviewing five published studies that measured and reported the ratio without the benefit of this or any such scale.

## 2. Proposed Scale for Mg Status Using the Ratio Between Serum Mg and Serum Ca

The obstacle to defining Mg status using serum Mg alone in general medicine and Mg research today may be overcome by using the ratio between serum Mg and serum Ca (either serum Mg/Ca or serum Ca/Mg). We present and test a proposed scale for discerning individual Mg status using this ratio.

The ratio between serum Mg and serum Ca can be expressed in four ways:

1. Serum Mg/Ca molar ratio (serum values in mmol/L or mEq/L)
2. Serum Mg/Ca weight ratio (serum values in mg/dL or mg%)
3. Serum Ca/Mg molar ratio (serum values in mmol/L or mEq/L)
4. Serum Ca/Mg weight ratio (serum values on mg/dL or mg%)

**Figure 1:**
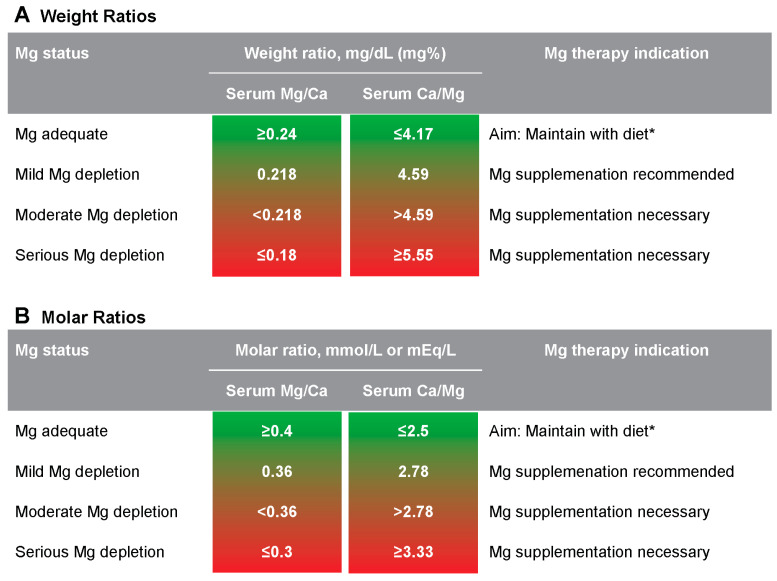
Serum Mg/Ca–Ca/Mg proposed scale to assess Mg status. To convert the weight ratio to the molar ratio, multiply by 1.65. To convert the molar ratio to the weight ratio, divide by 1.65. To convert Mg/Ca to Ca/Mg or vice versa, calculate the reciprocal (i.e., 1/value). * Maintain Mg adequacy with diet and add supplementation if necessary.

All four ratio calculations express the same physiological state, but they differ in analysis and valuation. Mg/Ca expressions increase with Mg adequacy and decrease with Mg depletion. Ca/Mg expressions are the opposite: they decrease with Mg adequacy and increase with Mg depletion. In addition, the numerical valuations for weight ratios are quite different from the molar ratios, even though they express the same physiological aspect. The serum Mg/Ca–Ca/Mg proposed scale for all four expressions is given in Figure 1.

## 3. Application of the Serum Mg/Ca–Ca/Mg Proposed Scale to Patient Case Studies

The serum Mg/Ca–Ca/Mg proposed scale reported here originates from case studies. The proposed scale was developed in private medical practice (Appendix B) and found helpful in defining Mg status for that setting (i.e., with individual patients). Clinical trials, such as those described in Section 4, give reliable, statistically tested information for groups of subjects. However, clinical trials cannot predict an individual patient’s response to any therapy, even when that therapy has been shown to be highly statistically significant in a well-designed study. For the physician or medical health care practitioner who makes necessary diagnostic and therapy decisions for one patient at a time, case studies, coupled with statistically rigorous clinical trial information, can be helpful.

The following four case studies demonstrate how the serum Mg/Ca–Ca/Mg proposed scale can be definitive of a Mg deficit, where serum Mg values alone could easily indicate no Mg problem to a vast majority of physicians and medical practitioners unfamiliar with Mg. All four case studies use the serum Mg/Ca molar ratio with the following proposed scale criteria for Mg status:

- ≥0.4: goal
- 0.36–0.399: sign of latent Mg depletion and Mg supplementation recommended
- 0.3–0.3599: definitive Mg depletion with doubtless supplementation needed
- ≤0.30: serious Mg depletion with high supplementation needed

### 3.1. Case 1—New Diagnosis: Should Mg Therapy Be Recommended?

A new female patient in her early 30s is Mg-naive and presents with high stress, high neuromuscular signs, dermal signs, tinnitus, and a long developmental history of psychological disorder (e.g., episodes of depression and anxiety). Her serum Mg level is 0.82 mmol/L. Some physicians and health care providers may view this level to indicate no problem with Mg [28], whereas some practitioners familiar with current Mg research would suspect a Mg deficiency because the patient’s serum Mg is <0.85 mmol/L [29]. Her Mg/Ca molar ratio is 0.33, which corresponds to serious Mg depletion on the proposed scale (Figure 1) and thus provides the diagnostic and therapeutic evidence for needed Mg therapy. Depending on this serum Mg measurement alone would result in a missed diagnosis of Mg deficit in the hands of most health care providers, leading to inadequate therapy.

### 3.2. Case 2—Impact of Mg Treatment: Should Mg Therapy Be Continued?

A female patient in her early 70s presents with neuromuscular signs, cardioneuropathy, slight peripheral neuropathy, and hypertension. She has been treated with Mg aspartate hydrochloric acid (15 mmol/d; recommended, 15–20 mmol/d) for more than 3 years. Her laboratory values show a serum Mg of 0.87 mmol/L, indicating no problem with Mg, and to stop supplementation because it is no longer necessary. However, a Mg/Ca molar ratio of 0.358 on the serum Mg/Ca–Ca/Mg proposed scale (Figure 1) suggests moderate Mg depletion, indicating that oral Mg therapy should be continued and the dose should be increased to optimize this patient’s Mg status. This optimal therapy was only evident with serum Mg/Ca.

### 3.3. Case 3—Impact in a Mg-Treated Patient with Diabetes: Should Mg Therapy (400 mg/d) Be Continued After a Year of Good Compliance?

A female patient with diabetes in her early 70s has shown credible good compliance with Mg therapy. She takes a proton pump inhibitor (PPI), and her diabetes is well controlled with metformin and dapagliflozin. However, she reports dizziness triggered by typical weather changes, cardioneuropathy, and peripheral neuropathy. Her general nutrition knowledge does not include potential Mg loss with diabetes and PPI use, so she wonders whether Mg supplementation has been effective and could be eliminated. Her serum Mg level is 0.83 mmol/L, indicating she should stop supplementation because it is no longer necessary per general medical opinion [28]; however, this level indicates to medical Mg experts that the Mg dosage should be increased [29]. The patient’s Mg/Ca molar ratio of 0.350 confirms she is experiencing Mg depletion (Figure 1), indicating that continuation and perhaps escalation of Mg therapy is needed.

### 3.4. Case 4—New Diagnosis in a Teen Patient: Should Mg Treatment Continue?

A male teen, pretreated with Mg citrate (300 mg/d), presents with a history of febrile cramps, no special neuromuscular signs, and slight obesity. His serum Mg level is 0.80 mmol/L, which suggests there is no Mg problem per general medical opinion, but indicates Mg deficiency (<0.85 mmol/L + history) per Mg experts. The patient’s Mg/Ca molar ratio is 0.316, indicating a serious Mg deficit according to the serum Mg/Ca–Ca/Mg proposed scale (Figure 1).

Use of the serum Mg/Ca proposed scale in these four case studies prevents missed diagnoses for Mg deficiency that could have led to inadequate therapy. Next, we consider whether this proposed scale can be useful in Mg research studies and clinical trials.

## 4. Application of the Serum Mg/Ca–Ca/Mg Proposed Scale to Published Research Studies

The serum Mg/Ca–Ca/Mg proposed scale described here was developed in private medical practice (see Appendix B) and has shown reliability in helping to diagnose Mg depletion in that setting (i.e., with individual patients). Such case studies, once crucial to the progress of medicine, are now often termed anecdotal evidence because they cannot give overall statistical assurance of effects for a given population. For the proposed scale to be truly useful, the case studies described in Section 3 must be coupled with statistically rigorous clinical trial information. It is thus expedient to determine whether the proposed scale can be applied in the research setting and to answer this question: Can the proposed scale’s use of the ratio between serum Ca and serum Mg be more definitive of Mg status than serum Mg measures alone? In our companion paper [30], we present our search strategy flow chart in gathering >40 published studies reporting this serum ratio using either Mg/Ca or Ca/Mg in molar or weight ratio expressions. None of these studies used any range measure with which to correlate their resulting ratios with a defined Mg status, so all are applicable to assessment by the proposed scale. The companion paper by Nelson et al. presents a proposed scale assessment for 10 of these published studies; future assessments of the remaining papers are planned. Here, we have selected five recently published studies from the companion paper collection with which to introduce the application of the proposed scale to published study results. We endeavored to select studies for a wide range of disease states and to include at least one using corrected serum Ca for evaluation with the proposed scale.

### 4.1. Research Study 1 by Guerrero-Romero et al.

Guerrero-Romero et al. (2022) [31] conducted receiver operating characteristic (ROC) analysis to evaluate the performance of the Mg/Ca weight ratio as a predictor of in-hospital mortality for patients with COVID-19. They measured the serum Mg/Ca weight ratio at admission for 1064 patients hospitalized with COVID-19 and separated the data into two groups: patients who recovered (i.e., were released from the hospital) and patients who died in the hospital. Mean serum Mg for the deceased group (1.91 ± 0.31 mg/dL) and the recovery group (1.97 ± 0.23 mg/dL) was adequate using the authors’ cutoff of 1.8 mg/dL (0.74 mmol/L) for hypomagnesemia. However, the mean serum Mg for both groups was <2.07 mg/d (0.85 mmol/L), which is an even narrower cutoff suggested for Mg adequacy [29].

Could the serum Mg/Ca–Ca/Mg proposed scale lend more specific information about the Mg status of the two groups studied by Guerrero-Romero et al. [31]? The ROC curve was drawn for Mg/Ca ratios to evaluate the performance of the serum Mg/Ca weight ratio as a predictor of in-hospital mortality. A cutoff point of <0.20 was identified to separate high-risk from low-risk patients, based on its sensitivity and specificity: 93.5% of deceased patients had a Mg/Ca weight ratio of <0.20, whereas only 14.7% of recovered patients had an Mg/Ca weight ratio of <0.2. The proposed scale value of <0.2 denoted a moderately depleted Mg status.

The area under the curve (AUC) of 0.521, which was slightly above the random guess point of 0.500, showed only slight predictive value for this Mg/Ca value for a death outcome in these patients. However, the sensitivity was high at 83%, suggesting that patients with COVID-19 with a serum Mg/Ca weight ratio of >0.2 at admission have a lower risk of in-hospital death than those with a ratio of <0.2. The multivariate logistic regression analyses showed a significant association between Mg/Ca <0.20 and discharge per death in the whole population (*p* = 0.0001), men (*p* = 0.001), and women (*p* = 0.001), and this association remained significant in the adjusted regression model for the whole population, men, and women. Because serum Mg levels alone showed general Mg adequacy for all patients in both groups, these findings suggest that the serum Mg/Ca molar ratio, more than serum Mg alone, may serve as a good warning of risk at admission for hospitalized patients with COVID-19, potentially enhancing the use of Mg treatment to lower the risk of in-hospital death from the disease.

### 4.2. Research Study 2 by Yang et al.

Yang et al. (2023) [32] measured the serum Ca/Mg molar ratio in healthy adults, seeking to define the ratio range for “health” in adults. The authors noted that Ca/Mg has been associated with several diseases, but there is no consensus on a reference range. They measured the serum Ca/Mg molar ratio in 337 adults aged >45 years who were deemed healthy in terms of body mass index, blood pressure, fasting plasma glucose, hemoglobin A_1c_, blood lipids, uric acid, hemoglobin, and heart rate; average plasma Mg was 0.88 mmol/L, certainly adequate by the latest serum Mg suggested reference range [29], and the prevalence of Mg deficiency was 6.66% taking 0.75 mmol/L as the lower cutoff limit. Yang et al. found that the serum Ca/Mg in these healthy individuals ranged from 2.36 to 3.66 (i.e., from certainly Mg adequate to seriously Mg depleted on our proposed scale). More specifically, they reported that 2.5% of participants with the lowest Ca/Mg (at 2.36) were certainly Mg adequate according to the proposed scale, and the 2.5% with the highest Ca/Mg (at 3.66) were seriously Mg depleted; the remaining 95% of participants were within those Mg status extremes. Both the mean (2.90) and median (2.84) serum Ca/Mg weight ratios for these healthy subjects fell into the Mg-depleted categories of our proposed scale.

The application of our proposed scale to the results of Yang et al. [32] shows that most of these presumably healthy adults (deemed so by plasma Mg concentration plus several common non-Mg risk factors for both heart disease and type 2 diabetes) were, in fact, Mg-depleted, some seriously so. If their Mg status was not corrected via diet, Mg therapy, or both, these individuals were at risk for developing diseases that come from an uncorrected latent Mg deficit in which Mg body stores are in active physiological depletion—a risk not evident with commonly used clinical measures nor common serum Mg reference ranges alone. Following these subjects in a prospective study to see how many developed heart disease, diabetes, or other manifestations of Mg depletion would be a great test of the proposed scale reported here, as well as the diagnostic value of the serum Ca/Mg ratio if such a study were deemed ethical.

### 4.3. Research Studies 3 and 4 by Meisel et al.

Meisel et al. (2005, 2016) [33,34] conducted cross-sectional and follow-up studies of patients with periodontal disease, in which they used the serum Mg/Ca molar ratio. They found that the serum Mg/Ca molar ratio was more relevant for analysis of periodontal disease progression than serum Mg alone.

The initial cross-sectional study was conducted in 2005; ref. [33] measured three aspects of periodontal disease (probing depth, attachment loss, and fewer remaining teeth) for 4290 subjects categorized by serum Mg/Ca molar ratio quartiles. The quartile ranges, along with serum Mg/Ca–Ca/Mg proposed scale assessment levels, are shown in Table 1.

Meisel et al. showed lesser degrees of periodontal distress with higher serum Mg/Ca for all three measures in subjects aged 40–80 years, seemingly in a dose-dependent manner (see Figure C in Meisel et al. [33]). The serum Mg/Ca levels driving these results compare well with the Mg status categories designated by our proposed scale (see Table 1).

Following 2432 of these subjects for a mean of 5 years, Meisel et al. [34] used serum Mg/Ca in three categories rather than four quartiles for their analysis: Q1, Q2–Q3 combined, and Q4. Their reported mean serum Mg/Ca for these three categories showed three levels of Mg status by the proposed scale’s Mg assessment for this 5-year follow-up: serious Mg depletion (Q1), moderate Mg depletion (Q2–Q3), and adequate Mg to mild Mg depletion (Q4) (Table 2).

In addition to the serum Mg/Ca categories, the 2016 follow-up analysis of periodontal disease progression by Meisel et al. [34] also divided subjects by inflammatory status according to C-reactive protein levels: CRP ≤ 3 indicated no systemic inflammation, whereas CRP > 3 indicated the presence of systemic inflammation. Most subjects (78–89%) had CRP ≤ 3. For these subjects, the 5-year follow-up showed an improved attachment level for all Mg/Ca categories, with most improvement in Q4 (i.e., Mg adequate to mild depletion, according to the proposed scale). For the 11–22% of subjects with CRP > 3, attachment level also improved, also seemingly in a dose-dependent manner with serum Mg/Ca category, but to a lesser degree than for those with CRP ≤ 3.

In subjects with CRP > 3, higher serum Mg/Ca (i.e., higher Mg adequacy, according to the proposed scale) was protective against tooth loss in a dose-dependent manner. However, for the majority of subjects with CRP ≤ 3, tooth loss was essentially even across the quartiles, with an insignificant trend toward greater tooth loss in Q4 than Q1 and Q2–Q3. These differences in tooth loss with inflammation demonstrate the importance of interaction between inflammatory state and Mg status, as defined by the serum Mg/Ca molar ratio in periodontal disease progression.

In both studies, Meisel et al. [33,34] found better attachment gains with high serum Mg, increased attachment loss with high serum Ca, and both solitary serum measures within a close range for all subjects. They also found that serum Mg/Ca was more relevant than single serum cations alone. In addition, the 2016 follow-up study [34] showed that the inflammation state, interacting with Mg status denoted by Mg/Ca, is also an important factor in the progression of periodontal disease.

### 4.4. Research Study 5 by Jiang et al.

#### 4.4.1. Overview: Impact of Corrected Serum Ca Methodology on Proposed Scale Usage

Jiang et al. (2024) [35] investigated the Ca/Mg molar ratio in individuals with acute coronary syndrome (ACS). Can the serum Mg/Ca–Ca/Mg proposed scale be useful for Mg status in such cases? In brief, these authors’ use of corrected Ca rather than total serum Ca showed inconsistent results; use of the proposed scale with the ratio we recalculated with total serum Ca showed consistent results and good diagnostic potential of both the ratio and the proposed scale.

#### 4.4.2. Detailed Analysis of Jiang et al. Shows Expected Multivariate but Unexpected Univariate Results

Let us review this study in more detail. Jiang et al. [35] asked, “Might Ca/Mg at admission be more informative of cardiovascular disease outcomes than serum Mg alone?” They retrospectively analyzed the clinical data of 1775 hospitalized patients presenting with ACS with the serum Ca/Mg molar ratio measured at admission. Multivariate analysis of Ca/Mg quartiles showed that higher Ca/Mg at admission (i.e., higher chance of Mg depletion) was correlated with increased risk of postoperative major bleeding and new-onset atrial fibrillation, as expected. This led Jiang et al. to conclude that “serum Ca/Mg levels at admission were significantly associated with adverse outcomes in patients with ACS, and was a reliable predictor of poor prognosis in acute coronary syndrome (ACS) patients” [35]. However, univariate analysis showed that in-hospital mortality was *inversely* related to serum Ca/Mg, because those in the lowest quartile (i.e., least chance of Mg deficit) had the highest mortality rate. This trend is opposite to what we would expect with a severe coronary condition such as ACS, characterized by both high urgency and high mortality rates.

Given their ACS diagnosis, one would expect all of the patients in the study by Jiang et al. [35] to be at least somewhat Mg deficient. However, using the serum Mg/Ca–Ca/Mg proposed scale, all four quartile Ca/Mg values reported range definitions falling within the proposed scale’s adequate Mg category (all <2.75; Table 3). The mean reported serum Mg for quartiles Q2, Q3, and Q4 fell within the commonly used serum Mg normomagnesemic range (i.e., 0.75–0.96 mmol/L [28]), whereas the Q1 mean serum Mg was slightly hypermagnesemic by this measure (at 0.97 mmol/L) but normomagnesemic by others [36,37,38,39]. Does this mean that serum Mg, the Ca/Mg ratio, the proposed scale, and, indeed, Mg status itself are not relevant at all to these patients’ ACS condition, let alone to its severity? We must look at the data more closely.

#### 4.4.3. Possible Use of “Corrected” Serum Ca (for Albumin) in Ratio Calculation May Explain Unexpected Results

Using reported mean serum Mg and Ca values to calculate serum Ca/Mg ratios for this study, all quartiles were deemed “adequate” Mg using the proposed scale (see Table 4). Digging deeper, what can the data from Jiang et al. [35] tell us? This study did not describe the methodology used to determine serum Ca, so it is possible that these serum Ca values are actually corrected Ca values (i.e., these reported serum Ca values were “corrected” using a simultaneous serum albumin measure via a standard formula).

#### 4.4.4. Recalculation of Ca/Mg Ratio Using “Uncorrected” Serum Ca Resolves Unexpected Results of Jiang et al. Univariate Results

Mean serum albumin values were reported for each quartile because they showed a significant protective factor against mortality risk (see Figure 2 in Jiang et al. [35]) in the univariate analysis we must consider for its unexpected result (see Table 4 and Section 4.4.2 above). With these serum albumin values, we are thus able to calculate a mean uncorrected serum Ca (i.e., total serum Ca), using the reported mean Ca values and the mean serum albumin values for each quartile with the following formula:

Uncorrected total Ca (mmol/L) =  Corrected Ca (mmol/L) + 0.8 × [40 − Serum albumin (g/L)]

We can then recalculate the mean serum Ca/Mg for each quartile mean using this uncorrected total serum Ca value. (See our calculations in Table A1 in Appendix A.)

The result (Table 4) gives this univariate analysis a more expected and understandable picture that reinforces the multivariate findings of this study: Q1, with its highest all-cause in-hospital mortality rate, now shows the highest Ca/Mg value and a serious Mg depletion status via the proposed scale. Succeeding quartiles Q2 and Q3 show descending ratios and low mortality rates, both in moderate to serious Mg depletion status. Q4 shows the lowest Ca/Mg value, the lowest mortality rate, and a mild to moderate Mg depletion status via the proposed scale. Thus, Q1 (the most Mg-depleted according to the proposed scale) shows the highest mortality of all four quartiles, and Q4 (the proposed scale’s least Mg-depleted of all four quartiles) showed the lowest mortality of the four quartiles—the expected results.

However, we were only able to recalculate the ratios in Jiang et al. [35] using the means for serum Ca, serum albumin, and serum Mg. The true value of this study’s data lies in the recalculation of each subject’s serum Ca/Mg using total serum Ca rather than corrected Ca and a revised statistical analysis. Revisiting the data in such a manner could then redistribute the subjects into true Ca/Mg quartiles for redux of the univariate as well as multivariate analyses.

Based on our review of the study by Jiang et al. [35], the serum Mg/Ca–Ca/Mg proposed scale requires the use of total serum Ca and total serum Mg in the calculation of the ratio to have true diagnostic and analytical value.

## 5. Discussion

The serum Mg/Ca–Ca/Mg proposed scale highlights the potential of serum Ca and Mg ratios to better ascertain Mg status than serum Mg alone. Its benefits and limitations are discussed next.

### 5.1. Limitations of Serum Mg: Rationale for Commonly Used Serum Mg Cutoffs Defining Hypomagnesemia in Use Today

Mg homeostasis is now known to include three physiological processes in the regulation of serum Mg: (1) absorption level in the gut; (2) reabsorption level in the kidneys; and (3) transfer of Mg from body stores, the most important being bone and muscle [18]. Thus, when gastrointestinal absorption and kidney reabsorption of Mg are both at their physiologically adapted maximum, translocation of Mg body stores can come into play [19]. It is safe to assume that patients with hypomagnesemia are in an active physiological process of body-store Mg depletion (more so with the greater degree of hypomagnesemia) and thus in need of Mg therapy. However, currently, four varying cutoff levels for hypomagnesemia are recommended or in active use (see Table 5).

The lack of agreement as to which serum Mg cutoff defines hypomagnesemia renders the research literature on Mg cumbersome, difficult to review, and filled with potential underassessment of physiological Mg deficit in human subjects. Indeed, the percentage of subjects deemed in need of Mg differs greatly when different cutoff points are used [26,27]. Debates as to which of these cutoffs is best for determining hypomagnesemia are ongoing. In the meantime, active physiological depletion of Mg body stores continues to be undiagnosed in patients, both ambulatory and hospitalized. We suggest a change in approach: Use of the serum Mg/Ca (Ca/Mg) ratio with the proposed scale reported here may enhance diagnosis of these patients and subjects using a more precise definition of Mg deficit than serum Mg alone, benefiting both medical practice and Mg research. Our initial findings here and in our companion paper [30] suggest the worthiness of further study.

### 5.2. Can the Serum Mg/Ca–Ca/Mg Proposed Scale Values More Precisely Determine Physiological Mg Status than Definitions of Hypomagnesemia?

In general medicine and Mg research today, the obstacle to defining Mg status using serum Mg alone may be overcome by using serum Mg/Ca (or serum Ca/Mg). We presented and tested a proposed scale for discerning individual Mg status, using this ratio as used in individual medical practice and published research.

#### 5.2.1. Development of the Proposed Scale in Mg/Ca Molar Expression

The proposed scale reported here, using the serum Mg/Ca molar ratio, was developed by B. von Ehrlich, MD, in private medical practice over 15 years (see Appendix B). The proposed scale was found to reliably enable the assessment of patient Mg status and recommend Mg therapy. The four case studies described in Section 3 of this review showed that serum Mg/Ca can be definitive of a Mg deficit or mild depletion status, where serum Mg values alone could easily indicate no Mg issue to a vast majority of physicians and medical practitioners, given the wide range of definitions of hypomagnesemia in practice today.

#### 5.2.2. Expansion of the Proposed Scale to Ca/Mg as Well as Mg/Ca (Both Molar and Weight Ratios)

The proposed scale’s expression for Ca/Mg as well as Mg/Ca in both weight and molar ratios was expanded by A. Rosanoff to enable its application to published Mg research studies and trials. Using three of the four proposed scale expressions in published trials and studies, this introductory review found the clarification of Mg status for a wide range of health and disease conditions: determination of health [32], definition of Mg status in acute cardiovascular disease [35], risk of mortality in hospitalized patients with COVID-19 [31], and periodontal disease progression [33,34]. We suggest and expect to see more applications of the proposed scale for several other existing studies that measured and reported ratios of serum Mg and serum Ca. Further reviews of other studies appear in our companion paper [30].

### 5.3. Proper Use of the Proposed Scale

Serum Mg and serum Ca values can be measured easily and inexpensively, and they can be used to calculate serum Mg/Ca or serum Ca/Mg. Using the proposed scale directly requires both serum Ca and serum Mg measurements in identical units (i.e., both mg/dL or mg%, both mEq/L, or both mmol/L). This is especially needed to allow meaningful comparison of ratio outcomes from different laboratories. When faced with diverging units, a clinical laboratory or research team can calculate accurate conversions to use the proposed scale’s parameters. For use in Mg research publications, the methodology requires a full and accurate description of the derivation of the ratios. Both measures need to be total serum Mg and total serum Ca, and use of a corrected serum Ca will give erroneous results (see Section 4.4 for a review of Jiang et al., 2024 [35]). Authors need to be precise in describing their methodology, and editors and reviewers must require clear definitions as to whether the reporting is in weight or molar ratio and Mg/Ca or Ca/Mg.

### 5.4. Naming Mg Status Proposed Scale Categories

Mild Mg depletion on the proposed scale can be understood as “just sufficient” in describing Mg status. Therefore, a Mg depletion category, to our understanding, should be denominated from <0.36 for the Mg/Ca molar ratio, >2.78 for the Ca/Mg molar ratio, <0.218 for the Mg/Ca weight ratio, and >4.59 for the Ca/Mg weight ratio. We refer to the level just beyond this sufficiency cutoff as “moderate Mg deficit or depletion.”

Many medical conditions are termed moderate to severe, and the term *severe* was considered for this proposed scale. During the writing of this manuscript, we considered this question: Is “severe” the right adjective for the fourth level of the proposed scale? When we reviewed the 2005 and 2016 studies by Meisel et al. [33,34], we visualized ambulatory patients sitting in dental chairs showing the fourth level of Mg depletion; in the 2023 study by Yang et al. [32], we thought of “healthy” subjects also showing the fourth level of Mg depletion. Thus, these individuals certainly are a different population from hospitalized patients with severe Mg deficiency receiving intravenous Mg sulfate for survival. Therefore, we designated the fourth level as “serious” Mg deficiency rather than “severe.”

### 5.5. Limitations

The purpose of this review is to introduce the serum Mg/Ca–Ca/Mg proposed scale with its four expressions, to present case studies, and to begin testing its applicability to group analysis in human Mg research for the first time. For this purpose, we selected published studies using only serum Ca and serum Mg values and case studies of mostly adults. Further study is needed to explore this proposed scale’s applicability with plasma, whole blood, and ionized values for Mg and Ca, along with these questions: Can this proposed scale be informative in animal studies, or is this proposed scale species-specific? Is it appropriate for use in subjects with chronic kidney disease, pregnant and lactating individuals, children, or in the wide range of human diseases with a Mg component? Many areas of study remain to fully utilize the diagnostic and research value of this proposed scale and the physiologic Ca-to-Mg/Mg-to-Ca ratio. Further application of the proposed scale to existing studies, as well as statistical testing, is needed to determine whether the ratio between serum Mg and serum Ca can more precisely measure physiological Mg status than serum Mg alone. We begin this larger endeavor here and with our companion paper [30].

## 6. Conclusions

Considering global trends toward lower dietary Mg intakes and worldwide differences in established laboratory habits, we believe that all obstacles to diagnostic alert on Mg status must be avoided. We have presented a proposed scale of serum Mg and Ca ratios as a diagnostic tool for Mg status in four expressions (i.e., Ca/Mg or Mg/Ca, molar ratios, or weight ratios). This study shows that the proposed scale is applicable to subjects in Mg research studies as well as individuals in medical practice. This proposed scale is a relatively inexpensive, highly available measurement that is worthy of further study. The proposed scale may give more diagnostic value for Mg status than serum Mg alone but this needs further research.

For proper use, the proposed scale needs both Ca and Mg measures in the same units, such as mg/dL or mg% (for weight ratios) and mmol/L or mEq/L (for molar ratios). Total serum Ca and/or Mg measures should NOT be corrected for serum albumin.

Authors need to be precise in describing their methodology when utilizing the serum Mg/Ca ratio. In addition, editors and reviewers must require clear definitions as to whether the reporting is the weight or molar ratio and Mg/Ca or Ca/Mg.

This review tested the proposed scale using ratios for total serum Mg and total serum Ca values in human adults. Future study is needed to determine whether this proposed scale is appropriate for other populations (chronic kidney disease, pregnancy, lactation, pediatrics) and with whole blood or ionized measures of serum Ca and Mg.

## Figures and Tables

**Table 1:** Quartiles of serum Mg/Ca molar ratios used to assess three aspects of periodontal disease (N = 4290).

	Quartile			
	Q1	Q2	Q3	Q4
Serum Mg/Ca molar range	≤0.298	>0.298–≤0.318	>0.318–≤0.342	>0.342
Proposed scale category of Mg status	Serious Mg depletion	Moderate to serious Mg depletion	Moderate Mg depletion	Mg adequate to moderate Mg depletion

Data are from Meisel et al. (2005) [33].

**Table 2:** Baseline mean serum Mg/Ca molar ratios for quartiles used to assess follow-up periodontal disease (N = 2432).

	Quartile		
	Q1	Q2–Q3	Q4
Serum Mg/Ca (molar), mean ± SD	0.28 ± 0.01	0.32 ± 0.02	0.38 ± 0.04
Proposed scale category of Mg status	Serious Mg depletion	Moderate Mg depletion	Mg adequate to mild Mg depletion

Data are from Meisel et al. (2016) [34].

**Table 3:** Reported results suggesting Mg adequacy for patients with acute coronary syndrome for both serum Ca/Mg (with presumed corrected Ca methodology) and serum Mg alone (N = 1775).

	Quartile			
	Q1	Q2	Q3	Q4
**Reported values for serum Ca/Mg (serum Ca corrected for albumin)**				
Reported range of quartile’s Ca/Mg molar ratio (using corrected Ca values)	<2.373	2.374–2.517	2.518–2.675	>2.676
Proposed scale category of reported quartile’s Mg status	Mg adequate	Mg adequate	Mg adequate	Includes Mg adequate
Mg status using reported mean serum Mg alone *				
Reported mean serum Mg of quartile (from Table 1 in Jiang et al., 2024 [35]), mmol/L	0.97	0.90	0.87	0.79
Mg status by wider serum Mg range (0.75–1.05 mmol/L) [36] *	Mg adequate	Mg adequate	Mg adequate	Mg adequate
Mg status by narrower serum Mg range (0.85–0.95 mmol/L) [29] *	Hypermagnesemic (>0.95 mmol/L)	Normomagnesemic	Normomagnesemic	Hypomagnesemic (<0.85 mmol/L)

Data are from Jiang et al. (2024) [35]. * Assessing Mg status using serum Mg alone is hampered by the many varying reference ranges for serum Mg in use [24].

**Table 4:** Comparison of reported serum Ca/Mg molar ratios using reported mean serum Mg and Ca (latter corrected for albumin) and revised ratios using reported mean serum Mg and recalculated mean total serum Ca (i.e., not corrected for albumin) *.

	Quartile			
	Q1	Q2	Q3	Q4
**Calculated serum Ca/Mg using reported mean serum values**				
Mean serum Ca/Mg calculated using reported mean Mg and Ca (latter corrected for albumin) values (see Table 1 in Jiang et al., 2024 [35])	2.23	2.46	2.57	2.90
Proposed scale category of Mg status	Mg adequate	Mg adequate	Mg adequate	Moderate to serious Mg depletion
Recalculation of serum Ca/Mg using total serum Ca (uncorrected for albumin *)				
Revised Ca/Mg using uncorrected serum Ca *	3.60	2.94	2.89	2.80
Proposed scale category of Mg status	Serious Mg depletion	Moderate to serious Mg depletion	Moderate to serious Mg depletion	Mild to moderate Mg depletion

Data are from Jiang et al. (2024) [35]. * See the revision calculation in Appendix A.

**Table 5:** Serum Mg cutoff points currently defining hypomagnesemia.

Currently Used Definitions of Hypomagnesemia (Serum Mg Cutoff)				Citation(s)
Mg Status	mmol/L	mEq/L	mg/dL (mg%)	
Low	<0.60	<1.8	<1.46	Ehrenpreis et al., 2022 [37]
Moderately low	<0.70	<1.4	<1.7	Al Alawi et al., 2018 [38]; de Baaij et al., 2022 [39]
Moderate	<0.75	<1.5	<1.82	Lowenstein & Stanton, 1986 [28]; Ahmed et al., 2019 [40] (citing Elin, 1987 *Clin Chem* [2])
High	<0.85	<1.7	<2.06	Elin, 2010 [4]; Costello et al., 2016 [41]; Micke et al., 2021 [29]; Rosanoff et al., 2022 [24]

## Data Availability

Data sharing not applicable.

## Abbreviations

The following abbreviations are used in this manuscript:

ACS	acute coronary syndrome
AUC	area under the curve
Ca	calcium
CRP	C-reactive protein
Mg	magnesium
PPI	proton pump inhibitor
Q	quartile
ROC	receiver operating characteristic

## Appendix A

**Table A1:** Recalculation of serum Ca/Mg molar ratio: calculation methodology for converting corrected serum Ca values (reported) to total serum Ca values (uncorrected).

	Quartile			
	1	2	3	4
Reported mean serum Ca, presumed corrected for albumin (mmol/L) *	2.16	2.21	2.24	2.29
Reported serum albumin (g/L) *	38.33	39.46	39.66	40.10
Mean serum Ca mmol/L (uncorrected) calculated using the following: Total Ca (mmol/L) = Corrected Ca (mmol/L) + 0.8 × [40 − Serum albumin (g/L)]	3.496	2.642	2.512	2.21
Revised Ca/Mg using uncorrected serum Ca	3.60	2.94	2.89	2.80
Proposed scale category of Mg status	Serious Mg depletion	Moderate to serious Mg depletion	Moderate to serious Mg depletion	Mild to moderate Mg depletion

* Values are from Table 1 in Jiang et al. (2024) [35].

## Appendix B

**Table A2:** Development of the proposed scale to assess Mg status using serum Ca and Mg ratios.

Timeframe	Medical Practice of B. von Ehrlich, MD, Kempten (Allgau), Germany
1990s	Magnesium (Mg) depletion as a clinical syndrome characterized by variable syntropies of symptoms was observed in our medical practice in conjunction with our review of the literature. Opinion-leading researchers propagated neglecting serum testing because of low specificity, resulting in misleading conclusions by pseudo-normal Mg serum results (based on a still widespread but false Mg normal value of 0.70 mmol/L).
2000–2010	To improve our clinical expertise, we considered and wrote about the influence of experience in clinical diagnosing of Mg depletion. We stated that clinical Mg diagnosis is highly dependent on specific Mg expertise and that the lack of experience leads to a high rate (>90%) of undiagnosed Mg cases. (We compared our results with the test frequency and the results of the largest German and European Laboratory, Schottdorf) [42].
	Experiments with even short-time (24 h) loading tests of Mg revealed them as impractical for the highly prevalent Mg depletion problem. We determined that Mg loading to determine the degree of adequacy was an erroneous approach.
	Clinical practice showed that even if we increased or set serum Mg cutoff values to 0.8 or 0.85 mmol/L, a number of patients with false normal results remained when compared with clinical diagnosing and the course of patients.
2010–2015	We observed a *swamb* (i.e., sponge) effect (translocation of Mg stores in a Mg-depleted state) of serum Mg measured in closely watched patients. For example, “normal serum magnesium” in a Mg-depleted stressed person (heart rate typically high, etc.) with Mg supplementation and second testing showed lower serum Mg with better condition of the patient “sponge”—published at Bologna Mg world congress).
	Single papers reporting the Ca/Mg ratio (coming primarily from Ca researchers) induced our careful observation of serum Ca as well as serum Mg. Observationally, we detected that many Mg-supplemented patients with initial high serum Ca (within normal range) showed decreased serum Ca under Mg supplementation. This was much more frequent than the literature narrative paralleling of Mg and Ca depletion.
	Patients as well as physicians in this digital age tend to think numeric values are more reliable. If we cannot achieve the necessary clinical widespread experience necessary for diagnosing Mg depletion without the clinical gold standard of the Mg loading test, we must use a parameter that is practical, inexpensive, and suggestive (i.e., understandable by patients).
	Mg and Ca act antagonistically in many—but not all—functions. Using this physiological fact and using the psychological fact that explaining to patients must be simple and logical, we concluded that with a low Mg/Ca ratio—in Mg depletion—we can simply explain (and show) that increasing Mg will normalize the pathological status.
	Since 2012, we have paralleled serum Mg determination with serum Ca determination (both in mmol/L) and calculated the Mg/Ca ratio individually in our practice, organized by an additional graph in our electronic patient documentation. In this early phase of measuring and calculating serum Mg/Ca ratios for our patients, along with our observations and experienced clinical documentation of Mg depletion, we found that 0.36 might be a suitable serum Mg:Ca ratio cutoff for Mg adequacy (i.e., less urgent Mg therapy) if no clinical symptoms are detectable.
	As time and experience progressed between 2012 and 2015, we used no definite cutoffs but rather ranges, producing a color graph showing red, yellow, and green zones with which we recommended achieving the green area (0.36–0.4 mmol/L or above for serum Mg/Ca ratio). This range showed reduced or low incidence of Mg depletion symptoms in clinical examination. This color graph has been a successful tool to convey the message to patients, raising compliance.
	In 2015, we began initial work combining measures of patients’ pulse wave velocity (AIX) with their serum Mg/Ca molar ratio. We found that high AIX paralleled with lower Mg/Ca (i.e., with Mg deficit).
2016–2020	By 2016, this work paralleling high AIX with lower serum Mg/Ca ratios in patients with mild cognitive impairment and dementia allowed us to conclude that “a serum Mg/Ca quotient of ≥0.4 is optimal, with 0.36–0.28 being too low” [43].
	This finding was presented in Rome at the XIV International Magnesium Symposium “Magnesium in Health & Disease” Roma, Villa Malta, 23–24 June 2016. At this conference, A. Rosanoff saw our presentation and asked permission to expand our independent, case study-based research of an optimal Mg/Ca serum ratio into a Mg adequacy scale to be available in weight as well as molar ratios of both Ca/Mg and Mg/Ca.
2016–2024	We have continued to apply the scale to different cohorts of our medical office and sometimes presented findings at Mg conferences, at which we discussed its use in conditions such as first mild cognitive impairment or dementia (Rome 2015), dizziness (Bratislava 2022), COVID-19 and vaccination, metabolic syndrome, and celiac disease. We even tested the correlation of historic disease symptom reporting of individuals. For example, King Ludwig II of Bavaria (Neuschwanstein Castle building king who died in the 19th century) reported many Mg depletion symptoms, which correlated with same symptom cohorts of our office with a <0.4 serum Mg/Ca molar ratio, and could show with high probability (>90%) that King Ludwig II was suffering from Mg depletion, even though we are not able to measure his serum values.
2025	Collaboration began between A. Rosanoff of CMER and Dr. von Ehrlich to publish the proposed scale with case studies and examine published peer-reviewed studies. D. Nelson, PhD, expanded the application of the proposed scale to a large set of published studies.
	In summary, we have methodically used and tested the tool in full medical practice for 13 years (since 2012), continue its use, and recommend its further study to other physicians and researchers needing a tool to demark Mg deficit in individual humans.
